# *In vitro* regeneration and *Agrobacterium*-mediated genetic transformation of *Caragana korshinskii*

**DOI:** 10.48130/FR-2023-0014

**Published:** 2023-05-31

**Authors:** Bin Liu, Xiaorui Shang, Xuting Zhang, Wen Shao, Lulu Ren, Guojing Li, Mulan Zhu, Ruigang Wang

**Affiliations:** 1 Key Laboratory of Plants Adversity Adaptation and Genetic Improvement in Cold and Arid Regions of Inner Mongolia, Inner Mongolia Agricultural University, Hohhot 010018, China; 2 CAS Center for Excellence in Molecular Plant Science, Shanghai Chenshan Plant Science Research Center, Chinese Academy of Science, Shanghai 201602, China; 3 Shanghai Key Laboratory of Plant Functional Genomics and Resources , Shanghai Chenshan Botanical Garden, Shanghai 201602, China; 4 State Key Laboratory of Tea Plant Biology and Utilization/ Key Laboratory of Tea Biology and Tea Processing, Anhui Agricultural University, Hefei 230036, China

**Keywords:** *Caragana korshinskii*, Embryonic tip, Agrobacterium-mediated transformation, Organogenesis

## Abstract

*Caragana korshinskii* is a deciduous shrub with large eco-economic value and strong tolerance to abiotic stresses. However, the shortage of reliable genetic transformation technology severely hinders its research on stress tolerance mechanisms and stress-resistant gene mining and application. In this study, the embryonic tip of the *C. korshinskii* seedling was used as the initiating explant to get regenerated plant through the direct organogenesis pathway, which significantly shortened the culture cycle and set the foundation for investigation of *Agrobacterium*-mediated genetic transformation. Our results suggest that the embryonic tip possesses robust meristem capacity and is an efficient method for transgenic breeding. This research provides a technical basis for asexual reproduction, molecular breeding, and gene function investigation in *C. korshinskii* by establishing, for the first time, an effective *in vitro* regeneration system and an *Agrobacterium*-mediated stable genetic transformation system utilizing the embryonic tip of *C. korshinskii* as explants.

## Introduction

*Caragana korshinski* (*Caragana*, Leguminosae) is a deciduous perennial shrub (Supplemental Fig. S1) that is widely distributed across the desert and semi-desert areas in northwest China. It is an integral resource plant in vegetation restoration, ecological reconstruction, bio-energy, forage, and medicinal development, with strong adaptability and tolerance to the harsh environment^[1−3]^. Because of the important role of *C. korshinskii* in ecological protection and restoration^[4]^, related studies have mainly focused on its genetic diversity, ecological physiological characteristics, resource exploitation, and utilization^[5]^. In recent years, the molecular mechanism of *C. korshinskii*'s adaptation to adversity and its genetic improvement have attracted widespread attention. In addition, the rapid development of genomics in the last decade has significantly promoted the research and application of genetic transformation. As a shrub with long growth cycles, traditional breeding methods are time-consuming, inefficient, and largely unable to achieve rapid genetic improvement, while transgenic technology provides a feasible way to accelerate the genetic improvement of woody plants and cultivate excellent varieties. In view of the lack of a stable genetic transformation system, the key regulatory factors identified from the *C. korshinskii* genome can only be verified through allogeneic transformation of *Arabidopsis thaliana*, the model weed plant, which severely limits the ability to investigate the genetic regulation of the critical traits of *C. korshinskii* itself.

It is well-known that legumes are notoriously difficult to regenerate^[6]^, and thus far just a few legumes, such as *Glycine max*^[7]^, *Vigna unguiculata*^[8]^, and *Medicago truncatula*^[9]^ have established stable genetic transformation systems. Beginning in the 1970s, studies on *C. korshinskii* tissue culture concentrated on the use of stem segments, cotyledons, hypocotyls, cotyledon nodes, and other tissue fragments as explants, for the induction of adventitious shoots as well as micro-fast propagation and roots^[10−13]^. Although studies on organogenesis for the *in vitro* regeneration of *C. korshinskii* explants have been reported that generally suffered from low regeneration efficiency and poor reproducibility, and the inability to obtain transgenic plants using the above-mentioned scheme have largely limited the genetic improvement of *C. korshinskii* by genetic engineering.

Researchers have demonstrated that the type of beginning explants and the age of the seedlings were among the most influential aspects of *in vitro* culture regeneration^[14]^. In the process of tissue culture, it is crucial not only to select acceptable explants, but also to ensure a consistent genetic background, and to choose the size of the explant, the tissue site, the growth stage, and manipulation are especially important. In general, the smaller the explant the lower the survival and reproduction rates; concurrently, the initial explant material should contain a large population of cells with high regeneration characteristics to differentiate under hormone induction. Due to its high differentiation rate, rapid regeneration, low mutability, short cycle time, low additional material, regeneration unaffected by seasonality, ease of *Agrobacterium* infestation, and reduced chimerism in obtaining transgenic seedlings, the embryonic tip is an ideal explant material for legumes^[15]^.

In recent years, it has been demonstrated that the embryonic tip is a sensitive explant where the regeneration system has been successfully applied and optimized for genetic transformation research, particularly in soybean and cowpea. Liu & Wei introduced for the first time an *Agrobacterium*-mediated genetic transformation system for soybean embryonic tips, with a transformation efficiency of 8.6%−11.7%^[7]^, and discovered that the regeneration frequency of embryonic tips was roughly double that of cotyledon nodes. Researchers have established numerous cultivars of soybean embryonic tip transformation systems by improving the parameters impacting embryonic tip regeneration and transformation systems^[16−20]^. At present, the cowpea organogenesis pathway is studied mostly using cotyledon nodes, but this technique is incompatible with *Agrobacterium*-mediated transformation^[21]^. To address the inefficiency of transgenic selection and *in vitro* shoot regeneration in cowpea, researchers developed a rapid, stable, flexible, and efficient transformation system using embryonic tips as explants and a CRISPR/Cas9 mediated genome editing system with a 68.6% genome editing efficiency, obtained non-chimeric heritable transgenic seedlings^[8,22]^. It can be seen from the report that the application of embryonic tip in legumes have been continuously improved, and using the embryonic tip as an initial explant is superior to other explants, but the occurrence of adventitious buds is highly variable due to many factors including plant genotype, plant growth regulators, and basal media. Although the performance of embryonic tip regeneration varies among species because of the presence of numerous influencing factors, there is still much space for the development of *C. korshinskii* embryonic tip *in vitro* regeneration and genetic transformation system, and we have not observed any research related to *C. korshinskii* embryonic tip as the starting explant for *in vitro* culture and *Agrobacterium*-mediated genetic transformation so far.

It is difficult to perform *in vitro* culture of *C. korshinskii* because of problems such as its fragile and tiny embryonic tips, smooth surface, difficult to handle, and difficulty in separation. Therefore, how to produce an initial explant is a crucial step in establishing its genetic transformation. In this study, we attempted to 'enlarge' the embryonic tips by adding exogenous hormones, and then used the 'enlarged' embryonic tips as explants for *in vitro* regeneration and genetic transformation. Then, we established a rapid, stable, and efficient regeneration system through the direct organogenetic pathway, and tested the transformation efficiency with *β-glucuronidase* (GUS)^[23]^ and RUBY^[24]^ reporter gene. On this basis, the Dehydration responsive element binding protein (DREB) encoding gene, named *CiDREB1C* by our group previously^[25]^, had been transformed into *C. korshinskii*, and the regenerated transgenic plant had finally been obtained. It is the first report to establish an efficient regeneration system and stable genetic transformation system for *C. korshinskii*.

## Materials and methods

### Plant materials and culture conditions

Seeds of *C. Korshinskii* were collected for embryonic tip explant isolation from wild populations in Siziwang County (41°25′48 N, 111°41′24 E), Ulanqab City, Inner Mongolia Autonomous Region, in mid-August 2020. Basic MSB5 medium (MS salt B5 vitamin)^[26]^ used in all experiments and supplemented with inositol 0.1−0.2 g·L^−1^, 3% sucrose, and 6% agar. The pH of all media was adjusted to 5.80 ± 0.05 before autoclaving at 121 °C for 17 min, culture temperature was 25 ± 2 °C, light intensity 2,500−3,000lx, and the light/dark period was 8 h/16 h.

**Figure 1:**
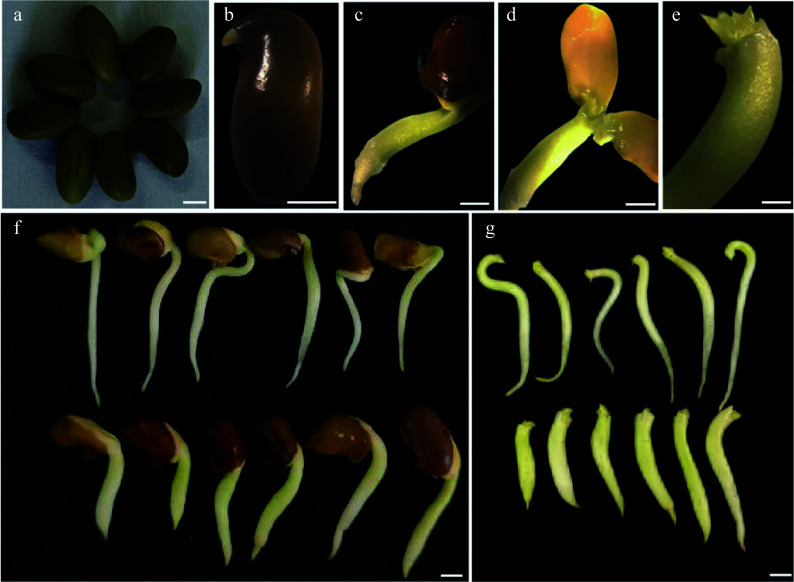
The enlarged embryonic tip explants after pre-treatment of the seeds of *C. korshinskii*. (a) Freshly sterilized seeds of *C. korshinskii*. (b) - (d) Embryonic tip explant preparation. (e) The 'enlarged' explants. (f), (g) Comparison of the phenotype of the germinating seeds between untreated (the upper panel of the photographs) and cytokinin-treated ones (the lower panel of photographs). Bar = 2 mm.

To establish a sterile system, healthy seeds without insect wounding and no black spots were selected (Fig. 1a) and were continuously rinsed under tap water for 30 min, and then washed with detergent for 3 min; the testa wiped with a 75% alcohol-soaked cotton wrap, and then the seeds soaked in 75% alcohol and were slightly shaken for 2 min under aseptic conditions; the seeds were washed 2−3 times with sterile water, and then were disinfected with 10% NaClO for 20 min while the flask was continuously shaken; the seeds were then washed with double sterilized water 5−6 times, the surface of the seeds was blotted with sterile filter paper, and the seeds (with the hilum downward) were inoculated into seed germination medium (MSB5) for further study.

### Regeneration of embryonic tip *in vitro*

**Figure 2:**
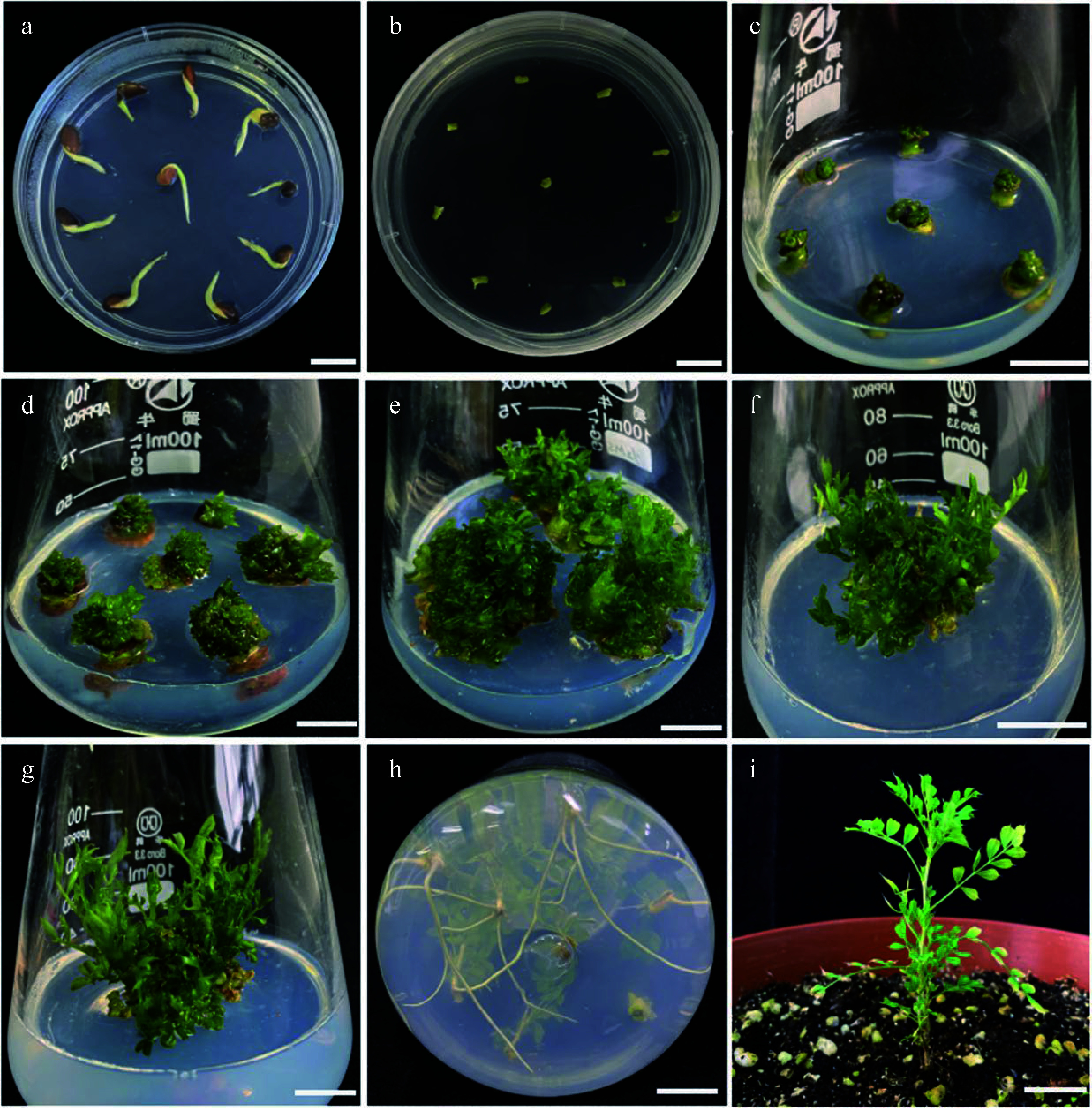
The complete process of the regeneration system from embryonic tip of *C. korshinskii in vitro*. (a) Pre-treated germinating seeds of *C. korshinskii*. (b) The embryonic tips begin to turn green and to enlarge. (c) Adventitious buds induction after priming. (d) Successful induction of adventitious buds. (e) 'Storm-like' adventitious buds. (f) First step elongation of the adventitious buds. (g) Second step elongation of the adventitious buds. (h) Adventitious roots induction. (i) Healthy seedlings after transferring into soil for 40 d. Bar = 1 cm.

After about 4 days of germination, seeds were transferred to pre-cultivating MSB5 medium with 6-Benzylaminopurine (6-BA) 1 mg·L^−1^ and cultured for approximately 1 week to get germinating seeds with short and thick radicles. The germinating seeds were removed from the seed coat and dissected longitudinally along the cotyledon axis to remove the cotyledons, plumules, and radicles, then obtained the 'enlarged' apical starting explants of about 2 mm in length (Fig. 1e), and finally, the 'enlarged' embryonic tips inoculated into MSB5 adventitious shoot induction medium (supplemented with 0, 1, 2, 3, 4, 5, and 6 mg·L^−1^ 6-BA respectively). Approximately 30 d following inoculation, the rate of adventitious shoot induction was assessed, and the optimal shooting induction medium was chosen. The clustered shoots were then subcultured once (12 d/time) to produce 'storm-like' adventitious buds (Fig. 2e).

The 'storm-like' adventitious buds were then transferred to MSB5 with different concentrations of plant growth regulators including 0.1−2 mg·L^−1^ 6-BA and 0.01−0.2 mg·L^−1^ α-Naphthaleneacetic acid (NAA) and cultured in a stepwise elongation design. In the first phase , the elongation culture lasted around 25−30 d, and the adventitious bud elongation was measured when the adventitious buds exhibited a considerable tendency to elongate. In the second phase, the concentration of the plant growth regulator was lowered suitably throughout the elongation and culture stage; and then, after once more of a subsequent culture, the elongated and robust sprouts were obtained.

For root induction, the elongated bush shoots with a height of more than 2.5 cm was separated into single seedlings and placed in MSB5 medium with 0, 0.5, 1, 2, or 3 mg·L^−1^ NAA respectively. After culturing for four weeks, the adventitious root induction rate and growth state were determined, and the optimal rooting induction medium was chosen. Plant acclimatization and transferring to pots according to the following method: select the robust plants and remove the caps from the culture bottles, filling them with 0.5−1 cm of water, loosely replacing the caps (not tightened), and leaving the culture bottles at room temperature under natural light for 2−3 d. The plantlets removed from the culture bottles, and the associated media cleaned, then ready for transfer into the soil. The optimal transferring medium consisted of peat soil, perlite, and vermiculite at a ratio of 3:1:1. Before transferring, the soil with the pot was immersed in water to make it completely moist, and after transferring, the plastic bag used to keep moisture for the plantlets. When the humidity was insufficient, the plantlet's surface was sprayed with water using a spray bottle. The plastic bag was removed after the seedlings grew stronger and then the seedlings were maintained and taken care of normally. After 5 weeks, the survival rate of the rooted plant was counted.

### *Agrobacterium *strains, inoculation and co-culture

The *Agrobacterium tumefaciens* strain GV3101 (which had been used successfully in *Agrobacterium*-mediated transient gene expression in *C. intermedia* by our group)^[25]^*,* the plant expression vector pCambia1305.2 with Hygromycin (Hyg) plant screening marker used in this study provided by our laboratory, and the plant expression vector pCanG-HA with Kanamycin (Kan) plant screening marker kindly offered by Prof. Qi Xie, the Institute of Genetics and Developmental Biology, Chinese Academy of Sciences. The pDR5: RUBY vector with Hyg plant screening marker was kindly offered by Prof. Yubing He, the State Key Laboratory of Crop Genetics and Germplasm Innovation, College of Agriculture, Nanjing Agricultural University.

*Agrobacterium *GV3101 harboring *GUS*, *RUBY*, and *CiDREB1C* plasmids kept at −80 °C were removed and put on ice, a small number of unfrozen blocks were inoculated using a sterilized gun tip into containing 50 mg·L^−1^ Kan^+^ and 50 mg·L^−1^ gentamicin (Gen^+^) to LB liquid medium (with 10 g·L^−1^ NaCl + 10 g·L^−1^ Tryptone + 5 g·L^−1^ Yeast extract, pH = 7.0), the bacteria were shaken at 28 °C with 200 rpm culture until the log growth phase was reached. Three hundred μL of the above bacterial solution was transferred to 50 ml of the LB liquid medium with 50 mg·L^−1^ Kan^+^ and 50 mg·L^−1^ Gent^+^ for amplified culture, and shaken at 28 °C with 200 rpm until the optical density of the bacterial culture reached OD_600 _≈ 0.80. The well-grown *Agrobacterium* culture was transferred to a 50 ml EP tube and centrifuged for 10 min at 4 °C with 4,000 rpm, then the supernatant was removed, and the bacteria pellet was suspended in MSB5 liquid media.

Before transformation, embryonic tip explants were immersed in MSB5 liquid media supplemented with 3 mg·L^−1^ 6-BA for approximately 12 h on a rotary shaker with vibration (at a speed of 80 rpm). On sterile filter paper, the explants were blotted dry for roughly 3−5 min. The embryonic tips were then immersed in 50 ml of MSB5 re-suspension medium with an OD_600_ ≈ 0.50 and 200 μM acetosyringone (AS) for 10 min under vacuumed (0.1 MPa) conditions. The surface was then blotted dry with sterile filter paper and transferred to a co-culture medium (MSB5 + 3 mg·L^−1^ 6-BA + 200 μM AS, pH ≈ 5.8) for 2−3 d in the dark. (Note: The embryonic tip explants should be washed many times with sterile water before being transferred to the selection medium to limit the quantity of the residual Agrobacterium.)

### Optimal dose test of antibiotics

To determine the effect of antibiotics concentration on plant growth, embryonic tips, and regenerated seedlings were inoculated in MSB5 medium supplemented with different concentrations of cefalexin (Cef^+^) (100, 200, 300, 400, 500, 600 mg·L^−1^), Kan^+^ (10, 20, 30, 40, 50, 60 mg·L^−1^) and Hyg^+^ (3, 5, 10, 15, 20, 25 mg·L^−1^). After 2−6 weeks of incubation, the survival rate and growth status of embryonic tips and regenerated seedlings were counted to determine the optimal selection concentration of antibiotics.

### Resistance screening and plant regeneration

After washing the co-cultured embryonic tips 5−6 times with sterile water, absorbed in the extra water, and transferred the embryonic tips to the adventitious bud induction selection medium with 30 mg·L^−1^ Kan and 200 mg·L^−1^ Cef. After 3 d of bacteria static culture in MSB5 solid medium for recovery, they were transferred every 15 d. After the production of green-resistant buds, the yellow and white buds were removed and transferred to MSB5 solids medium with 40 mg·L^−1^ Kan and 200 mg·L^−1^ Cef for further screening. To avoid selection escape and chimeric events occurrence, after resistant plantlets showed a marked inclination to elongate, a medium with 60 mg·L^−1^ Kan was used to select the fertile transgenic plants once more. Resistant plants were allowed to grow to approximately 2 cm on the selection medium and were then excised and transferred to the rotting medium. All culture conditions were performed as described in the embryonic tip regeneration system.

### ***β***-glucuronidase staining

The histochemical staining for GUS followed the methods of Jefferson et al.^[23]^. Positively transformed tissues and the control were immersed in GUS staining solution and incubated overnight at 37 °C in the dark, after they were repeatedly immersed in 95% ethanol to fully remove chlorophyll, and photos were taken after complete destaining.

### Molecular identification of transgenic plants

Total DNA was extracted with the TSINGKE Plant DNA Extraction Kit (Universal) as outlined in the product instructions. For PCR identification, primers for 5-segment based on the whole sequence of the *RUBY* were designed. The detection of *CiDREB1C* was based on the primers specific to the CaMV35S promoter, the NOS terminator, and the kan gene. All the primers were designed using the program Primer premier 5.0. and the primer sequences were listed in Supplemental Table S1. PCR products were resolved by electrophoresis on a 1% agarose gel.

### Data processing

Three biological replicates were carried out in the experiments, and IBM SPSS (Statistical Product and Service Solutions) 19.0 was used to process the data. The significance of variations between the means of each treatment was assessed using Duncan's New Multiple Range Test, and P < 0.05 was used as the significant threshold. Graphs were made utilizing GraphPad Prism 8.0.2 and Adobe Photoshop CS.

## Results

### Amplified embryonic tip explant cultivation

Seeds of *C. korshinskii* germinated normally are very difficult to handle for explant preparation since the embryo tip is delicate, tiny, and generally quite smooth. Therefore, we pre-treated the explants: in brief, seeds that had germinated for approximately 4 d (Fig. 1b) were transferred to MSB5 pre-culture medium with 1 mg·L^−1^ 6-BA added and had cultured for approximately 1 week, and significant differences in appearance in the embryo tip (Fig. 1f, photos in the lower panel) were observed compared to those without cytokinin addition (Fig. 1f, photos in the upper panel). The pre-cultured seeds had short and thick embryonic axes, and the apical parts were also significantly thicker and tended to be 'enlarged' (Fig. 1c−e, & g, pictures in the lower panel), possibly as a result of cytokinin inhibition on the apical bud dominance and promotion on the increased cell division size.

### Embryonic tip *in vitro* regeneration system

After pre-treatment (Fig. 2a), 2 mm of the 'enlarged' apical explants were removed and inoculated into the adventitious shoot induction media, and then the explants have been kept for expansion for around 5 d (Fig. 2b). Granular protrusions could be seen to form on the surface of the expanded explants at around 20 d (Fig. 2c). Next, they were sub-cultured once to successfully generate adventitious buds (Fig. 2d). The adventitious bud induction rate was calculated statistically (Table 1), and it was discovered that with 6-BA concentration increased, the adventitious bud induction rate exhibited a tendency of initially increasing and then decreasing. When the concentration of 6-BA was 3 mg·L^−1^, the rate of adventitious bud induction reached 78%, the average number of buds per explant was around eight, and the buds were in a healthy growing condition. When the concentration of 6-BA was 4 mg·L^−1^, the induction rate of adventitious buds was 63%, and the average number of buds was around five. With the concentration of 6-BA increasing further, adventitious buds differentiation was hindered, while base browning and yellowish seedlings emerged. Therefore, 3 mg·L^−1^ 6-BA was the optimal hormone concentration for inducing adventitious buds from the 'enlarged' embryonic tip.

**Table 1:** Effect of different 6-BA concentrations on the induction rate of adventitious buds from embryonic tip explants.

6-BA concentration (mg·L^−1^)	Induction rate of adventitious buds (%)	Average number of adventitious buds
0	8.89 ± 0.02e	2.72 ± 0.25e
1	30.00 ± 0.07d	3.80 ± 0.18d
2	52.22 ± 0.05c	5.14 ± 0.38c
3	77.78 ± 0.07a	8.19 ± 0.29a
4	63.33 ± 0.06b	5.75 ± 0.24b
5	48.89 ± 0.07c	4.71 ± 0.25c
6	31.11 ± 0.02d	4.18 ± 0.17d
Values represent the mean ( ± standard error) of three independent experiments. Different letters of the same column indicate significant differences at *P* < 0.05.		

To improve the frequency of embryonic tip regeneration and obtain more regenerated buds, we observed the growth of adventitious buds at various stages (Supplemental Fig. S2) and noticed that after 20 d of culture, the embryonic tip had thickened, the base had enlarged, and occasionally 1−2 buds sprouted. The number of adventitious buds was between eight and 12 after 40 d of cultivation, and they formed tight clusters. After 50 d of culture, the embryonic tip expanded further, 'storm-like' adventitious buds (Fig. 2e), and the number of adventitious buds was between 26 and 31. However, as culture time prolonged further, the increasing rate of adventitious buds slowed down after 60 d, the leaves color of the bushy buds deepened, the buds felt more fragile, and the water-soaked appearance was severe. Therefore, the above-mentioned results indicate that the cluster buds required one subculture to produce 'stormy-like' adventitious buds. The local magnification images of the adventitious buds regenerated from the embryonic tips are shown in Supplemental Fig. S3.

Adventitious bud elongation requires both cytokinin and auxin, and in plant tissue culture, the predominant auxin used is naphthylacetic acid (NAA). For research on adventitious bud elongation, we induced longer shoots using a range of 6-BA and NAA combinations at various concentrations (Table 2). Except for the control, all other treatments were capable of making the adventitious shoots elongate. However, the elongation effect and growth status of the adventitious shoots varied greatly due to the various hormone levels. For example, after only one week in the medium without any added hormones, the yellow seedlings had already perished, and the adventitious buds showed no tendency to elongate. The greatest effect on elongation was achieved when 1 mg·L^−1^ 6-BA was combined with 0.1 mg·L^−1^ NAA, and some of the adventitious buds started to elongate after about 4 weeks of culture (Fig. 2f), and the adventitious bud elongation rate was 68% with good growth status. After subculture, a lot of calli formed at the base, and the shoots displayed various degrees of vitrification. At higher concentrations of hormone combinations, however, the elongation rate of adventitious shoots decreased, a large number of calli formed, and the elongated shoots displayed severe vitrification. Therefore, the hormone ratio of 1 mg·L^−1^ 6-BA and 0.1 mg·L^−1 ^NAA were only suitable for the one-step elongation of adventitious shoots that were transferred to MSB5 medium with 0.7 mg·L^−1^ 6-BA and 0.07 mg·L^−1^ NAA, the final elongated adventitious shoots were produced after 2 weeks (Fig. 2g).

**Table 2:** Effect of different combinations of 6-BA and NAA on the elongation of adventitious buds.

Treatments	Hormones and concentrations (mg·L^−1^)		Elongation rate (%)
	6-BA	NAA	
1	0	0	6.03 ± 0.04d
2	0.3	0.03	30.50 ± 0.07bc
3	0.5	0.05	41.43 ± 0.06b
4	0.7	0.07	59.49 ± 0.05a
5	1.0	0.10	67.92 ± 0.08a
6	2.0	0.20	26.11 ± 0.06c
Values represent the mean ( ± standard error) of three independent experiments. Different letters of the same column indicate significant differences at *P* < 0.05.			

A single shoot from the elongated buds (the shoot height > 2.5 cm) was separated and planted in an MSB5 medium with different concentrations of NAA to induce roots. The rate of adventitious root induction and the growth status of the resulting seedlings were then measured and calculated (Table 3). In this test, we found that the plant acclimatization and transferring to pots were difficult to survive if there were both massive calli growth and roots at the base of the stem. It is probably owing to the weak connection of the vascular tissue between the roots and shoots^[27]^. It was observed that 1 mg·L^−1^ NAA was the best concentration for inducing adventitious roots, and 3 cm of roots were visible after 15 d of induction. In addition, the adventitious roots developed directly from the vascular tissue when treated with this concentration of NAA. The rooted regenerating seedlings were obtained after over 30 d of culture (Fig. 2h), with a rooting induction rate of 79%. Calli developed readily at the root base when the NAA concentration was higher than the ideal one, and the induced adventitious roots were unhealthy and difficult to transfer successfully. Robust bottle seedlings were selected for nursling, and after 5 weeks of transferring, the survival rate of the rooted seedlings (Fig. 2i) was more than 95%.

**Table 3:** Effect of NAA concentrations on adventitious roots induction.

Treatments	NAA concentration (mg·L^-1^)	Number of explants	Rooting rate (%)
1	0	90	0d
2	0.5	90	41.11 ± 0.18bc
3	1	90	78.89 ± 0.15a
4	2	90	55.56 ± 0.14ab
5	3	90	28.89 ± 0.08c
Values represent the mean ( ± standard error) of three independent experiments. Different letters of the same column indicate significant differences at *P* < 0.05.			

### Antibiotic effects on non-transformed explants growth

**Figure 3:**
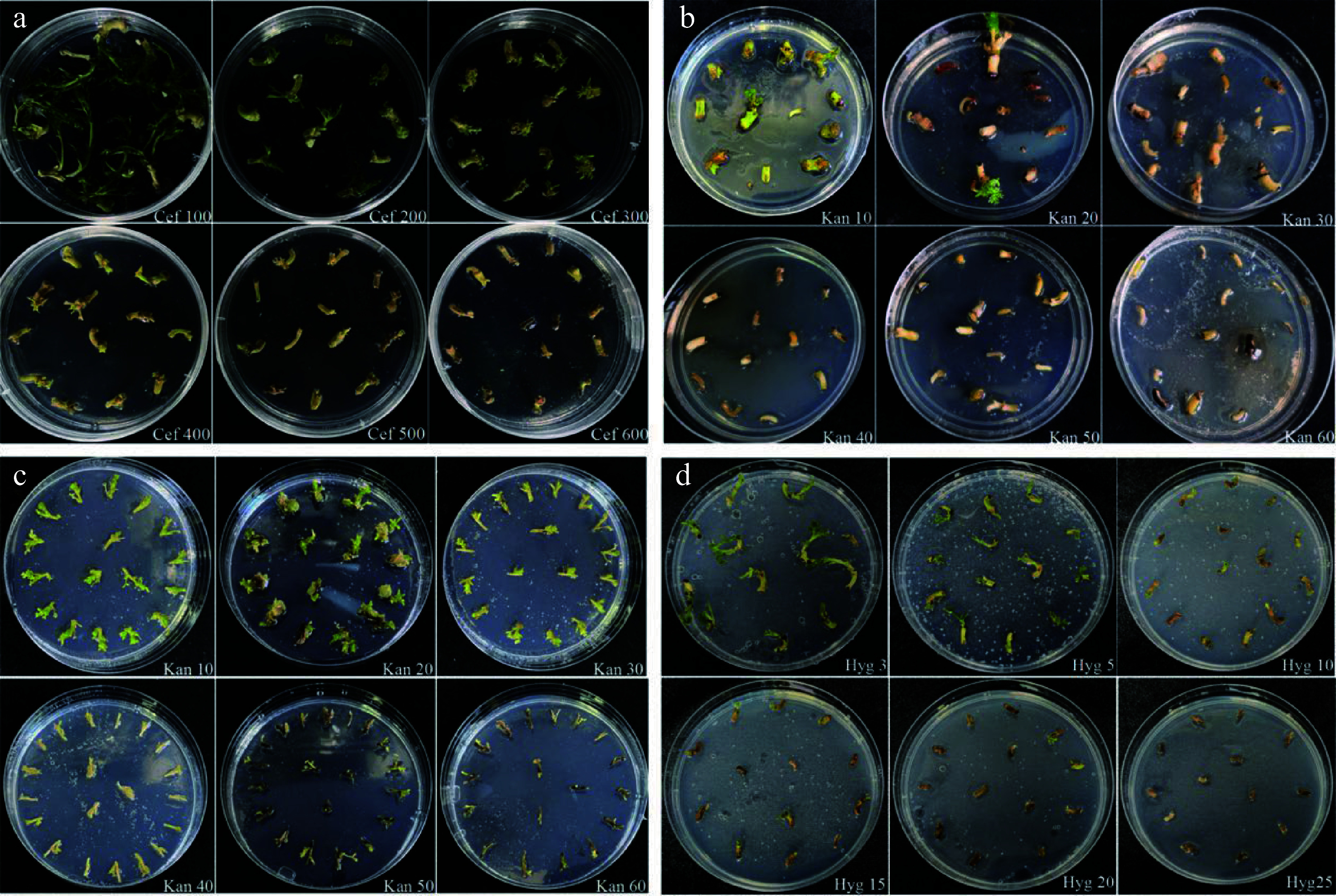
Effects of cefotaxime, kanamycin and hygromycin on growth of explants. (a) Growth status of the embryonic tips in response to different concentrations of cefotaxime. (b) Growth status of the embryonic tips after being treated with various concentrations of kanamycin. (c) Growth status of the regenerated shoots under gradient concentrations of kanamycin. (d) Growth status of the embryonic tips after being treated with various concentrations of hygromycin.

Appropriate concentrations of cefalexin have been proven to decrease the harmful effects of Agrobacterium on explants and to successfully inhibit *Agrobacterium *growth after co-cultivation^[28]^. Thus, we investigated how the embryonic tip was sensitive to cefalexin when establishing the *Agrobacterium*-mediated genetic transformation system. The results showed that embryonic tips were more sensitive (Fig. 3a) when the concentration of cefalexin was 300 mg·L^−1^ or higher, and the growth of the explants was obviously inhibited, yellowish and dead seedlings appeared at high concentrations of cefalexin. When the concentration of cefalexin was 200 mg·L^−1^, the sprouts grew slowly but in good status and could still develop into seedlings. So 200 mg·L^−1^ was determined as the optimal inhibitory concentration of cefalexin.

During *Agrobacterium*-mediated transformation, a step-by-step screening approach was used. After co-culture with *A. tumefaciens*, the embryonic tips should be transferred to a selection medium for culture. It is generally acknowledged that cells that survived the selective pressure would be positive transformants and that the screening antibiotics concentration should neither be too low to increase the possibility of false-positive plants, nor too high to inhibit the growth and differentiation of the transformed cells. Our results revealed that the sensitivity of different explants to kanamycin was not the same (Fig. 3b, c), and the frequency of explant regeneration decreased with the increasing kanamycin concentration. When the kanamycin concentration was 30 mg·L^−1^, the embryonic tip essentially stopped growing and the explants turned yellowish and died. Hence, the selection pressure of kanamycin concentration for the first step is 30 mg·L^−1^. To obtain more positive shoots, we increased the selection pressure in the latter stages. When the kanamycin concentration exceeded 40 mg·L^−1^, stem elongation was completely inhibited, and the explants perished. So the selection pressure of kanamycin concentration for the second step was determined to be 40 mg·L^−1^. To avoid selection escape and chimerism, 60 mg·L^−1^ Kan^+^ was used to select the fertile transgenic plants finally.

The embryonic tips were sensitive to hygromycin, and the survival rate decreased as the concentration increased. When the concentration of hygromycin was 3 mg·L^−1^, the growth of the explants was affected; when it was increased to 15 mg·L^−1^, the growth of the embryonic tips was severely inhibited and dead explants appeared; when the concentration of hygromycin reached to 25 mg·L^−1^, all the explants died. Therefore, 25 mg·L^−1^ was selected as the screening concentration of hygromycin.

### Genetic transformation and plant regeneration

**Figure 4:**
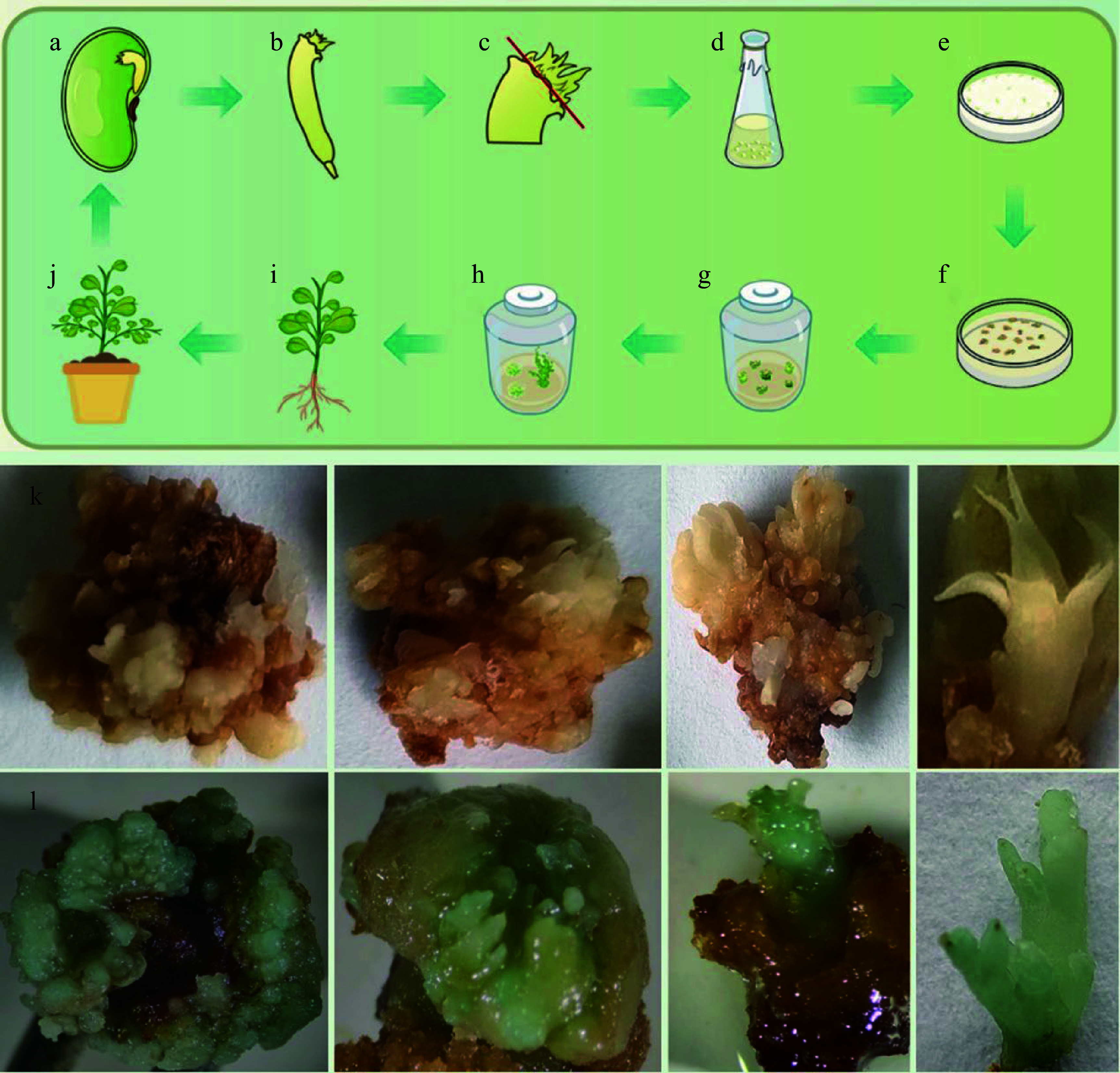
Schematic illustration of embryonic tip transformation system and GUS staining results in *C. korshinskii*. In the schematic of genetic transformation ((a)−(j), upper panel), the plumule is removed from the red line. (k) Pictures of GUS staining from the un-transformed calli and buds. (l) Pictures of GUS staining from the transformed calli and buds.

The process of genetic transformation of *C. korshinskii* by the recombinant construct carrying *CiDREB1C* gene (which bears the kanamycin resistance) and plant regeneration is shown in Fig. 4. Explants were co-cultured for 2 d (Fig. 4e), then were transferred to an MSB5 recovery medium and bacteriostatic culture with 200 mg·L^−1^ cef for 3 d. After which, an adventitious bud induction selection culture was performed; and 4−8 weeks later, kanamycin-resistant granular protrusions were observed (Fig. 4f). After 3−6 weeks, resistant adventitious buds were effectively induced (Fig. 4g), and after further transferring to adventitious bud elongation selection medium until shoots elongated (Fig. 4h); then they were transferred to rooting medium and adventitious roots were successfully induced in roughly 5 weeks. Transgenic plants had been obtained when plants with well-developed root systems were transferred into sterile soil (Fig.4i, j). Through rigorous screening, non-kanamycin-resistant buds ceased to grow, progressively turned yellow-brownish, and ultimately perished (Supplemental Fig. S4).

Using *GUS* histochemical staining that transformation efficiency was identified to demonstrate the event's reliability. The process of genetic transformation by the recombinant construct carrying the *GUS* gene (which bears the hygromycin resistance) and plant regeneration shown in the lower panel of Fig. 4. The histochemical verification of GUS activity was performed in presumed resistant transgenic buds, with the un-transformed buds serving as a negative control (Fig. 4k). Blue staining was observed during adventitious bud induction (Fig. 4l), indicating that the *GUS* gene had been stably expressed.

### Molecular identification of transgenic plants

**Figure 5:**
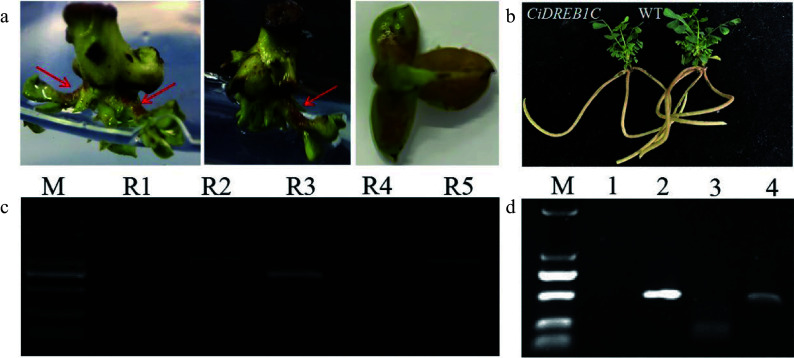
Accumulation of betalain in transgenic *C. korshinskii* buds and PCR detection of the *RUBY* and *CiDREB1C* genes. (a) Phenotype of RUBY-positive buds, as indicated by the red arrows. (b) *CiDREB1C* transgenic plants and the negative control (WT). (c) Eletrophoresis results of the PCR products from *RUBY* in the positive transgenic buds; Lane R1-R5, the PCR products from the five segments of *RUBY*. M, molecular weight marker (DL5000). (d) Electrophoresis results of the PCR products from *CiDREB1C* in the positive transgenic seedlings and wild type control; M, molecular weight marker (DL2000). Lane 1: negative control, PCR products for the *Kan* gene (lane 2), the NOS terminator (lane 3), and the CaMV35S promoter (lane 4).

Based on the initial success of transforming *GUS* reporter gene, we attempted to transform *RUBY* and *CiDREB1C* genes into *C. korshinskii* with an effort to set up a stable genetic transformation system. RUBY can function as a visual selection marker for transgenic events to identify transgenic-positive plants with an accumulation of reddish betalain. We achieved positive results in *C. korshinskii* which was consistent with that of other species^[8]^: the reddish color was observed in petioles and leaves (Fig. 5a) of the potential transformants, and further identification of the *RUBY* gene in transgenic buds were performed by extracting leaf genomic DNA for PCR amplification. The results revealed that the *RUBY* gene had been transferred into *C. korshinskii* successfully (Fig. 5c). To further test the efficacy of the genetic transformation system, the *CiDREB1C* gene was transformed into *C. korshinskii*, and primers specific to the *CaMV35S* promoter, the *NOS* terminator, and the Kan marker on the pCanG-HA vector were designed for PCR detection, and the un-transformed buds served as a negative control. The *CaMV35S* promoter was not amplified in the negative control, but the predicted bands were detected in the transgenic samples, as shown in Fig. 5d. The above results indicate that transgenic *CiDREB1C* plants can be produced with kanamycin selection, and all transgenic plants were viable following acclimatization and after transferring into the soil. Except for the dwarf phenotype of the transgenic *CiDREB1C* plants, there were no discernible differences compared to non-transgenic plants (Fig. 5b). Using embryonic tips as explants, our results show a stable transformation system for *C. korshinski* has been established. Even though the transformation efficiency is still low currently, the stable transformation system provides the technological foundation for future optimization.

## Discussion

According to the reports, domestic researchers had successfully built a direct adventitious shoot generation system for the *Caragana* Fabr.^[29]^. Nevertheless, the system is still in the initial stage^[30−32]^, and a stable and effective regeneration system is essential for genetic modification. It is widely recognized that the frequency of transgenesis in woody plants is low and time-consuming. In addition to their inherent specificity, the use of antibiotics and the integration of exogenous genes during genetic transformation can also influence subsequent differentiation and regeneration. For these reasons, research on transformation in the *Caragana* Fabr. has come to a standstill. The successful establishment of a genetic transformation system depends on a number of factors, among which the type of starting explants has the greatest influence. The cotyledon node^[33−35]^ and embryonic tip^[36,37]^ typically employed as initial explants in soybean transformation. The ability of explants to regenerate based on the type of explants, the method of cutting, the age of the explants, and the polarity of the material^[38]^, among other factors. The embryonic tip has a strong ability to differentiate less prone to genetic mutation, which makes it easier to handle later and an ideal receptor in the transgenic process, despite the somewhat increased labor of the preparation process in the early stages.

In order to overcome the difficulty of manipulating *C. korshinskii* embryonic tips, we pre-treated the initial explants and 'enlarged' the embryonic tips. It has been reported that the addition of a certain concentration of cytokinin to the medium during the acquisition of sterile seedlings considerably improves regeneration efficiency^[39]^, however, we did not observe any significant alterations in our study. It has shown that a certain concentration of cytokinin 6-BA can induce a high rate of adventitious bud differentiation^[40]^ and enhance the number of adventitious buds^[41]^. Our results showed that 6-BA plays a critical role in the induction of adventitious buds, and the average number of buds increases with the rise in 6-BA concentration, but when the 6-BA concentration is too high, inhibition on the formation of adventitious buds are also observed in our case, which is consistent with the previous results^[42]^.

Additionally, the type of medium impacted the reproduction of the explants^[43]^. MS medium was more suitable for bud elongation, whereas B5 medium was more conducive to bud differentiation, according to Diallo et al.^[44]^, which evaluated both MS and B5 media. Siva et al.^[45]^ were able to successfully transform the α-amylase inhibitor gene into cowpea seeds utilizing MSB5 medium and taking cowpea cotyledons as explants. We compared the four basal media, including MS, B5, MSB5, and WPM, and discovered that seed germination or non-growth in *C. korshinskii* on WPM medium, and when regenerated sprouts were subjected to subculture under the same hormonal conditions, growth stagnation, yellowish seedlings, and dead seedlings were observed, which may be owing to the low inorganic salt content of WPM medium and the imbalance of nutrient level, whereas there were no significant differences on MS, B5, and MSB5 media. According to the results of the previous findings, the MSB5 medium was selected as the basic medium^[7]^.

The high regeneration frequency is vital for transformation, and the use of an embryonic tip as the explant shows good regenerative characteristics, adventitious bud induction rate of the *C. korshinskii* regeneration system utilized in this study is 78%, and up to 12 adventitious buds can be produced from one explant, establishing the foundation for genetic transformation. At present, the embryonic tip regeneration system is mostly combined with the *Agrobacterium*-mediated transformation method. In this study, the embryonic tip of *C. korshinskii* was used for the first time as a transgenic receptor for studies on genetic transformation with transformation efficiency of about 0.6%. Unfortunately, the current stable transformation system is still inefficient. The resistance genes were verified by transient expression and heterologous expression in Arabidopsis, preventing the advancement of its gene editing technology. To overcome difficulty, a large number of transformation experiments are required. Therefore, improving transformation efficiency will be the focus of our future research, which aims to establish a genetic transformation system that is both highly stable and efficient.

Since the embryonic tip is relatively young and fragile, its growth capacity is diminished during the infection co-culture, therefore, the recovery and bacteriostatic culture is managed to perform after the co-culture, and the embryonic tip is transferred to the screening medium once its growth capacity has been restored. *GUS*, *RUBY*, and *CiDREB1C* were successfully introduced into *C. korshinskii*, however, no red color was observed in the stem segments of trans-RUBY plants, most probably because betalain was not substantially transported from the synthesis site to other tissues^[24]^ .The transgenic plants grew and matured as well as uninfected control plants, because cefalexin and kanamycin treatment was most probably responsible for the delayed growth of plants following genetic transformation.

In model plant *A. thaliana*, the flower dipping genetic transformation conveniently and greatly accelerated gene function investigation. However, it is not always working well or suitable for certain genes verification from *C. korshinskii.* For example*,* some transcription factors encoding genes did not contribute to drought tolerance when we transformed them into *A. thaliana*, although they were strongly induced in *C. korshinskii* when treated by drought stress. We speculated that these transcription factors might regulate a different set of downstream genes in *C. korshinskii* other than in *A. thaliana*. While we need a genetic transformation system of *C. korshinskii* to provide the solid evidence*.* In addition*,* genes involved in certain organs development, such as the stipular spines of *C. korshinskii*, could not be verified in *A. thaliana* either.

In addition, as a robust technique, gene editing technology has been used widely and successfully in plants, including many Legumes such as *Glycine max*^[46]^ and *Medicago sativa*^[47]^. We do believe that it is feasible to apply CRISPR-Cas to *C. korshinskii* with the appropriate genetic transformation system we established. However, we did not yet test it due to two reasons: one is the transformation rate is still low in our system; and that *C. korshinskii* has a juvenile period of 3−4 years, so it makes the selection of the homozygous edited offsprings more difficult. With further optimization of the transformation procedure, we expect that this genetic transformation system will be very helpful to verify its own gene's function in *C. korshinskii* instead of model plants.

## Conclusions

Using the embryonic tip as explant is an efficient method of transgenic breeding. We describe for the first time that the embryonic tip of *C. korshinskii* was utilized as the explant for *in vitro* regeneration and genetic transformation, and we established a stable and efficient *Agrobacterium*-mediated genetic transformation system, which has substantial advantages over previous research. The findings of this research provide a powerful technique foundation for the application of genetic engineering for the modification and improvement of *C. korshinskii*.

## SUPPLEMENTARY DATA

Supplementary data to this article can be found online.
