# Supplementary material for: In vitro regeneration and Agrobacterium-mediated genetic transformation of Caragana korshinskii
Source: For Res (Fayettev). 2023 May 31;3:14. doi: 10.48130/FR-2023-0014 (PMC11524263; doi:10.48130/FR-2023-0014)
Supplement: Supplementary file 1 — Supplementary data to this article can be found online. [file FR-2023-0014-S1.zip › 10.48130_FR-2023-0014-Suppl-FigureS3.docx]

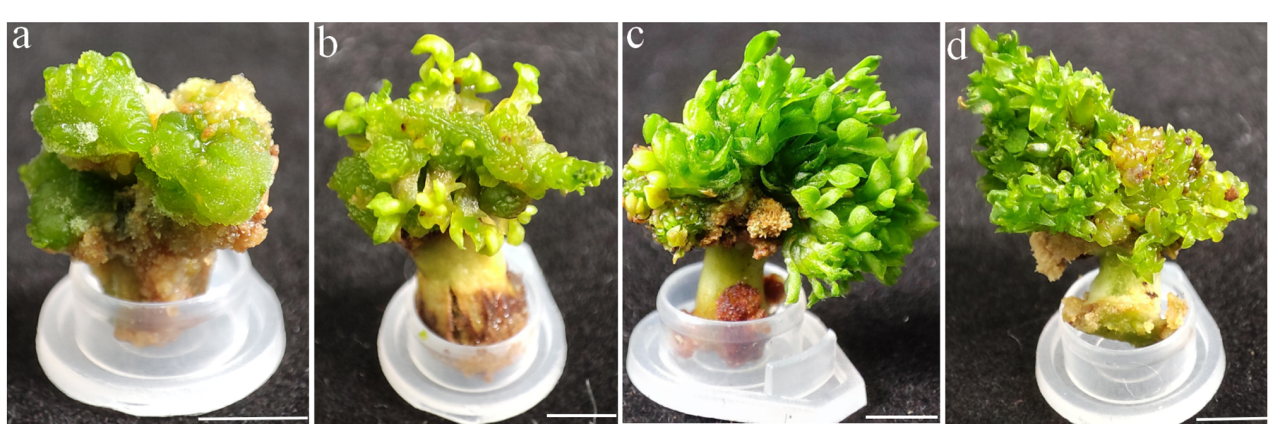


**Fig.S3 The local magnification images of adventitious bud regenerated from the embryonic tip.**

1. Granular protrusions were formed on the surface of the explants. (b) Initiation adventitious buds formation. (c-d) Successful induced adventitious shoots. bar=0.5 cm.
