# Supplementary material for: In vitro regeneration and Agrobacterium-mediated genetic transformation of Caragana korshinskii
Source: For Res (Fayettev). 2023 May 31;3:14. doi: 10.48130/FR-2023-0014 (PMC11524263; doi:10.48130/FR-2023-0014)
Supplement: Supplementary file 1 — Supplementary data to this article can be found online. [file FR-2023-0014-S1.zip › 10.48130_FR-2023-0014-Suppl-FigureS4.docx]

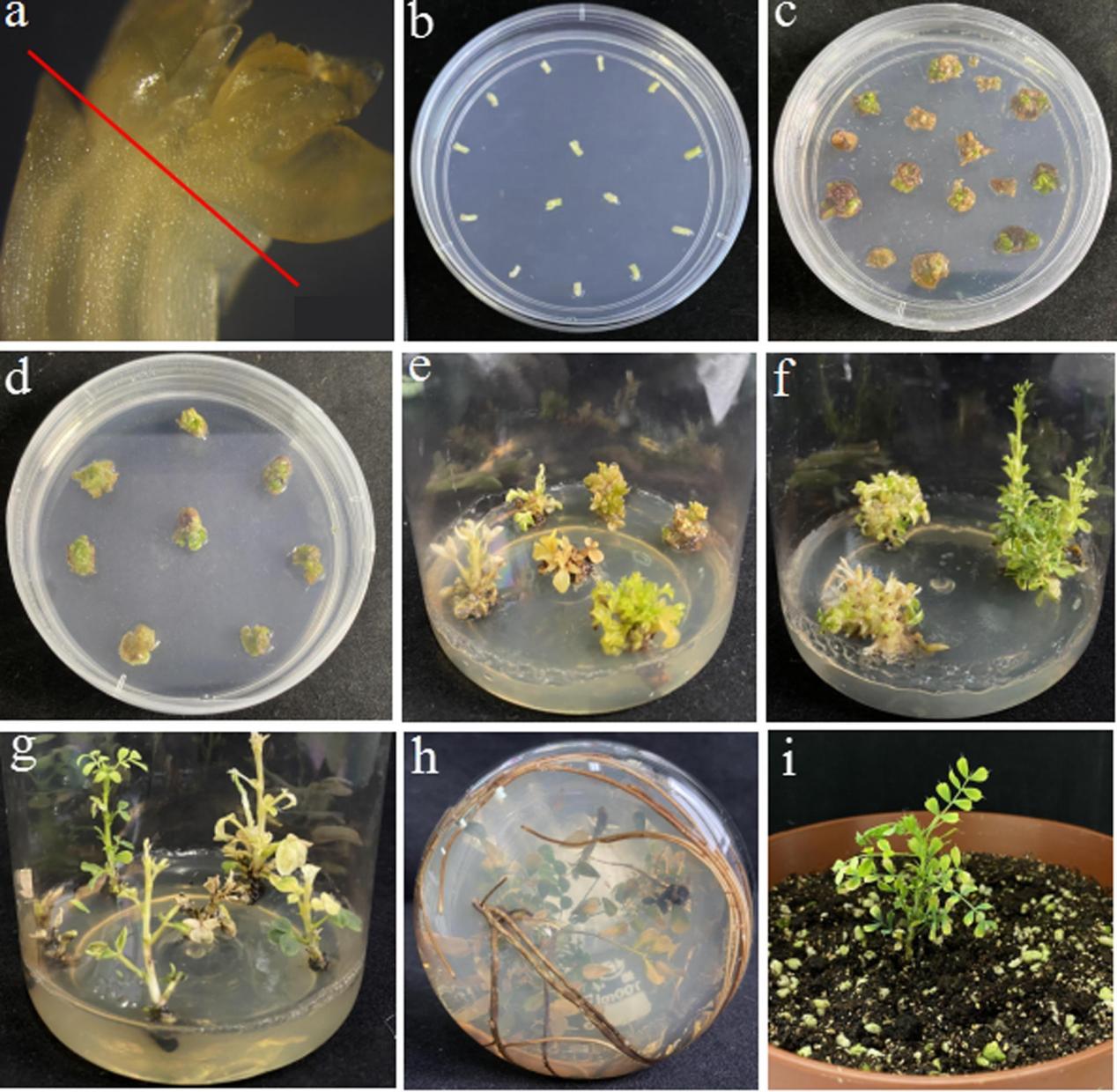


**Fig.S4 The genetic transformation process of *C. korshinskii* with *35S::HA-CiDREB1C* recombinant construct using embryonic tip as explants***.*

(a) Anatomical image of the embryonic tip starting explant. (b) Explants were co-cultured for 2 days. (c, d) The adventitious buds were transferred to the induction medium with kanamycin selection, and 4-8 weeks later, kanamycin-resistant granular protrusions were observed. (e) Resistant adventitious buds were effectively induced after 3 to 6 weeks. (f, g) The resistant adventitious buds were transferred to the elongation selection medium until shoots elongated. (h) Adventitious roots were successfully induced after roughly 5 weeks. (i) The transgenic plant was obtained.
