# Supplementary material for: In vitro regeneration and Agrobacterium-mediated genetic transformation of Caragana korshinskii
Source: For Res (Fayettev). 2023 May 31;3:14. doi: 10.48130/FR-2023-0014 (PMC11524263; doi:10.48130/FR-2023-0014)
Supplement: Supplementary file 1 — Supplementary data to this article can be found online. [file FR-2023-0014-S1.zip › 10.48130_FR-2023-0014-Suppl-TableS1.docx]

**Table S1 The sequence of primers used in this study**

| Primer names | Sequence | Product (bp) |
| --- | --- | --- |
| R-1F | CTGTTCTCCCAGCAGACCAC | 955 |
| R-1R | GACCTGCTTGATTTCCTCTTGG |  |
| R-2F | CCGTGTTCCAGGGCATCAT | 1016 |
| R-2R | TTCACGGTTGGCTTCACTGTC |  |
| R-3F | GACGCCAACGACCAGATGA | 698 |
| R-3R | GCGGGTAGAAGTGCTCTGGAA |  |
| R-4F | ACGGCACCTACCACTACAATCT | 1006 |
| R-4R | TTATGGTTCTCTGGGAAGCCTG |  |
| R-5F | GTGGGCTCTACAGGCGTGG | 1043 |
| R-5R | AGTTTGGCCTGCATGACGG |  |
| Ca MV35S-F | TAACAGAACTCGCCGTAAAG | 446 |
| Ca MV35S-R | ATAGTGGGATTGTGCGTCAT |  |
| NOS-F | GAATCCTGTTGCCGGTCTTG | 180 |
| NOS-R | TTATCCTAGTTTGCGCGCTA |  |
| KAN-F | CACTGAAGCGGGAAGGGACT | 488 |
| KAN-R | CGATACCGTAAAGCACGAGGAA |  |
