# Supplementary material for: In vitro regeneration and Agrobacterium-mediated genetic transformation of Caragana korshinskii
Source: For Res (Fayettev). 2023 May 31;3:14. doi: 10.48130/FR-2023-0014 (PMC11524263; doi:10.48130/FR-2023-0014)
Supplement: Supplementary file 1 — Supplementary data to this article can be found online. [file FR-2023-0014-S1.zip › 10.48130_FR-2023-0014-Suppl-FigureS1.docx]

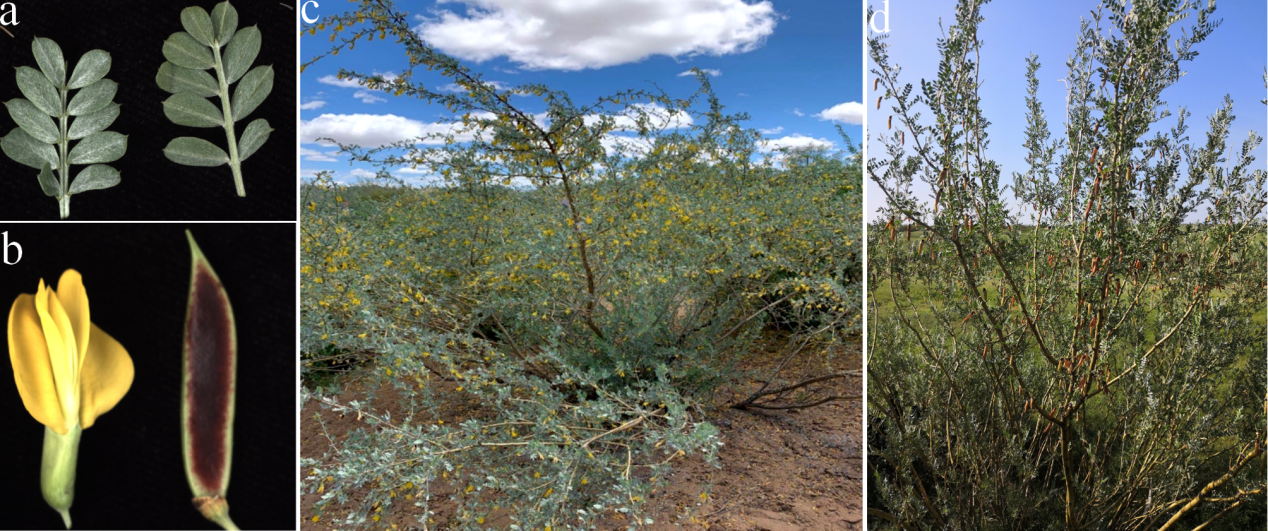


**Fig.S1 The images of *C. korshinskii* grown in natural environment.**

1. The left panel is the adaxial side of the leaf, and the right panel is the abaxial side of the leaf. (b) Flower (the left panel) and pod (the right panel) of *C. korshinskii*. (c) Blooming stage of *C. korshinskii.* (d) Pods bearing stage of *C. korshinskii.*
