# Supplementary figures and images for: In vitro regeneration and Agrobacterium-mediated genetic transformation of Caragana korshinskii
Source: For Res (Fayettev). 2023 May 31;3:14. doi: 10.48130/FR-2023-0014 (PMC11524263; doi:10.48130/FR-2023-0014)

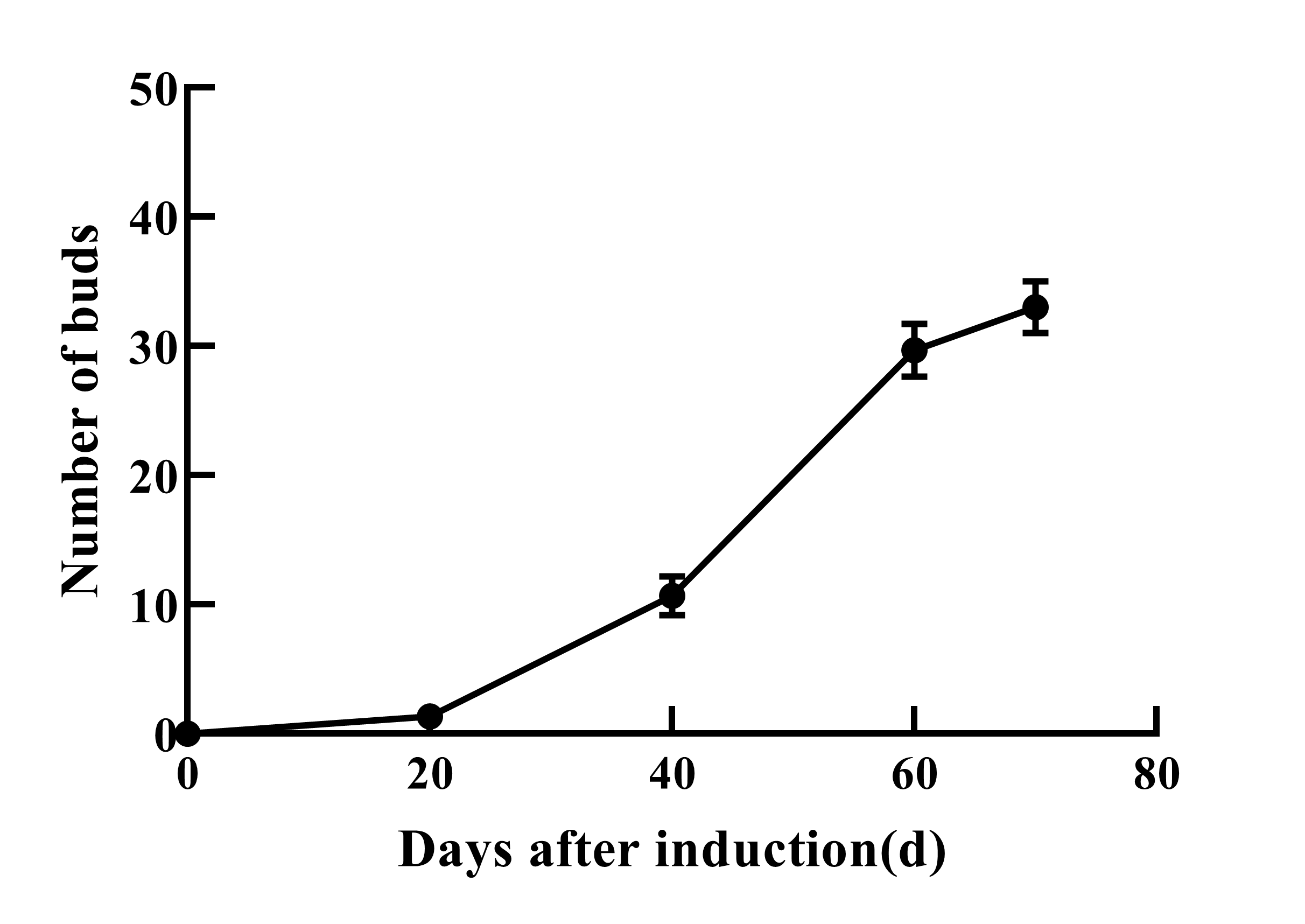


**Fig.S2 Effects of 6-BA on the number of adventitious buds at different induction time points.**

Supplement: Supplementary file 1 — Supplementary data to this article can be found online. [file FR-2023-0014-S1.zip › 10.48130_FR-2023-0014-Suppl-FigureS2.docx]
